# Recognition of Emotions in Mexican Spanish Speech: An Approach Based on Acoustic Modelling of Emotion-Specific Vowels

**DOI:** 10.1155/2013/162093

**Published:** 2013-07-10

**Authors:** Santiago-Omar Caballero-Morales

**Affiliations:** Technological University of the Mixteca, Road to Acatlima K.m. 2.5, 69000 Huajuapan de León, OAX, Mexico

## Abstract

An approach for the recognition of emotions in speech is presented. The target language is Mexican Spanish, and for this purpose a speech database was created. The approach consists in the phoneme acoustic modelling of emotion-specific vowels. For this, a standard phoneme-based Automatic Speech Recognition (ASR) system was built with Hidden Markov Models (HMMs), where different phoneme HMMs were built for the consonants and emotion-specific vowels associated with four emotional states (anger, happiness, neutral, sadness). Then, estimation of the emotional state from a spoken sentence is performed by counting the number of emotion-specific vowels found in the ASR's output for the sentence. With this approach, accuracy of 87–100% was achieved for the recognition of emotional state of Mexican Spanish speech.

## 1. Introduction

Emotion recognition has become an important research subject in human-computer interaction and image and speech processing [1]. Besides human facial expressions, speech has proven as one of the most promising modalities for the automatic recognition of human emotions [2]. Among the different applications of speech emotion recognition the following can be mentioned: psychiatric diagnosis, intelligent toys, lie detection, learning environments, and educational software [3].

Many approaches have been presented to recognize affective states based on specific speech features. Short-term features (formants, formant bandwidth, pitch/fundamental frequency, and log energy) and long-term features (mean of pitch, standard deviations of pitch, time envelopes of pitch, and energy) have been used for this purpose. Short-term features reflect local speech characteristics in a short-time window while long-term features reflect voice characteristics over a whole utterance [4]. Pitch/fundamental frequency (*f*_0_), intensity of the speech signal (energy), and speech rate have been identified as important indicators of emotional status [5–8]. Other works have shown that speech formants, particularly the first and the second, are affected by the emotional states [9, 10].

Acoustic speech features are represented with different methods, most of them related to speech recognition. Linear predictive coefficients (LPCs) have been used to represent the spectral envelope of a digital signal of speech in compressed form, using the information of a linear predictive model [11]. However, a problem faced with the LPCs for the process of formant tracking in emotion recognition is the false identification of the formants [8]. Mel-Frequency Cepstral Coefficients (MFCCs) provide a more reliable representation of the speech signal because they consider the human auditory frequency response [12]. Diverse works have used MFCCs as spectral features with significant results for emotion recognition [1, 3, 7, 13–16]. In [7] an alternative to MFCCs was presented in the form of short-time log frequency power coefficients (LFPCs).

Diverse classification methods are available for the recognition of emotions from the obtained speech features. In [16] high recognition accuracy was obtained with Support Vector Machines (SVMs) when compared with Naive Bayes and K-Nearest Neighbor. Other works have used Artificial Neural Networks (ANNs) [17–19] and Hidden Markov Models (HMMs) [13, 17, 19] with significant performance. In general, recognition tests with these methods are performed with long-term and short-term features which are obtained from speech corpora utterances with four or six emotions [8].

Most of the emotional speech databases cover the German and English languages (i.e., [19–23]). However, for the Spanish language, just few databases are known as presented in [8]. Particularly for the Mexican Spanish language, no speech database or developments in the field of speech emotion recognition are known.

In this work, the development of a Mexican Spanish emotional speech corpus following guidelines found in the literature for other languages is presented. In addition, an emotional speech recognizer is built with this corpus to test an emotion recognition approach. The approach consists in the phoneme acoustic modelling of vowels associated with each emotional state, considering that an emotional state is reflected as a tone variation of vowels. A standard phoneme-based Automatic Speech Recognition (ASR) system is built with HMMs, where different phoneme HMMs are built for the vowels associated with the considered emotional states. Estimation of the emotional state from a spoken utterance is performed by counting the number of emotion-specific vowels found in the ASR's output for the MFCC-coded utterance. With this approach accuracy of 87–100% was achieved for the recognition of the emotional state of Mexican Spanish speech.

This paper is structured as follows: in Section 2 the details of the Mexican Spanish emotional speech corpus (selection of emotional states, stimuli vocabulary, speech recording, phonetic and orthographic labelling, and acoustic features) are presented. Then, in Section 3 the details of the ASR system are presented while the results are presented, and discussed in Section 4. Finally, in Section 5, the conclusions and future work are presented.

## 2. Speech Corpus

One important resource for research in the emotion recognition field is the speech databases or corpora. Emotional speech data has been obtained from actors (simulated emotions) as in [22] and from spontaneous (non-acted) speech as in [19]. A more comprehensive list of speech databases with simulated and non-acted emotions is presented in [8]. Speech databases with simulated emotions are widely used for research given the similarities found between “real” and “acted” speech data [5].

In this work simulated emotional speech was obtained from Mexican non-professional actors and volunteers from the Cultural Center of the City of “Huajuapan de Leon” in Oaxaca, Mexico. As in [4], the text contents for the sentences of the corpus were written in a way to stimulate a speaker to speak in the specified emotions. About the number of emotions, in [24] the following 15 basic emotions were proposed: anger, fear, sadness, sensory pleasure, amusement, satisfaction, contentment, excitement, disgust, contempt, pride, shame, guilt, embarrassment, and relief. However, most of the emotional speech corpora consider four or six emotions [8]. In this work the following emotions were considered as defined in [25–28]: anger, happiness, neutral, and sadness. The details of the corpus sentences for the emotions and the speech acquisition process are presented in the following section.

### 2.1. Data Preparation

In Tables 1, 2, 3, and 4, the stimuli sentences for anger (“enojo”), happiness (“felicidad”), neutral (“neutro”), and sadness (“tristeza”) are presented, respectively. Ten sentences for each emotional state were designed, leading to a total of 40 sentences with 233 words (vocabulary of 140 unique words). The speech data was then obtained from four non-professional actors (two males, two females) from the local Cultural Center of the City of “Huajuapan de León” in Oaxaca, Mexico. Two additional volunteers (one male and one female) took part in the speech data collection. Thus, a total of six speakers (MS1-3, FS1-3) were considered for the emotional Mexican Spanish speech corpus, each one speaking the 40 sentences presented in Tables 1–4. This amount of speakers and speech data is similar to the corpus presented in [7] which considered six speakers for Burmese and Mandarin languages and ten sentences for each emotional state.

In this work, the emotional speech was recorded in WAV format with a sampling rate of 48,000 Hz and two audio channels. The software *WaveSurfer* was used for the orthographic and phonetic labelling of the speech data. Because the target language is the Mexican Spanish, special attention was paid to the pronunciation of the speakers as there are significant differences between the Spanish spoken in the South, Central, and North regions of Mexico.

For the definition of the phonetic repertoire for the labelling of the speech corpus (and development of the classifier) the Mexbet alphabet for the Mexican Spanish language [29] was used. An updated version of the alphabet, proposed by the Master in Hispanic Linguistics Cuetara [30], is shown in Table 5. This alphabet is specific for the Spanish spoken in the City of Mexico (Central Region) and the speakers for the emotional corpus had the associated pronunciation.

In addition to the Mexbet phonemes, Cuetara also proposed the inclusion of the archiphonemes /_D/, /_G/, /_N/ and /_R/ to define the neutralization of the following couples of phonemes: /d/-/t/, /g/-/k/, /n/-/m/, and /*ɾ*/-/r/ [30]. To represent the pronunciation of the sequence of phonemes /k/ and /s/ (as in “extra”), and the silence, the phonemes /ks/ and /sil/ were added. This led to a final alphabet of 28 phonemes for this work.

The spectral properties of vowel sounds have been found to be the best indicator of emotions in speech [27]. Also, work presented in [31, 32] reported on significant differences in vowels given the emotion used for their production. In this work for the identification of emotions, the following identifiers were added to the phonemes representing vowels: _e for “enojo” (anger), _f for “felicidad” (happiness), _n for “neutro” (neutral), and _t for “tristeza” (sadness). Thus, the vowels in the sentences for anger had the identifier _e, and the vowels in the sentences for sadness had the identifier _t. In Table 6 the frequency of emotion-specific vowels in the emotional stimuli from Tables 1–4 is presented.

**Figure 1:**
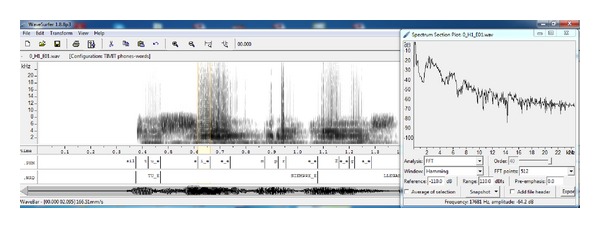
Spectrum section plot for the vowel “i_e” (/i/ with anger).

Once the emotional stimuli was recorded with the six speakers and the speech data was orthographically and phonetically labelled, a spectral analysis of the speech segments representing the emotion-specific vowels was performed. The software *WafeSurfer* was used for this task. As presented in Figure 1 a “Spectrum Section Plot” was obtained for each vowel in the speech corpus. The setting for the plot was: FFT Analysis, Hamming Window, Reference: −110.0 dB, Range: 110.0 dB, Order: 40, Pre-emphasis: 0.0, and 512 FFT points. The data points of each plot were saved in a text file by pressing the button “Export”.

**Figure 2:**
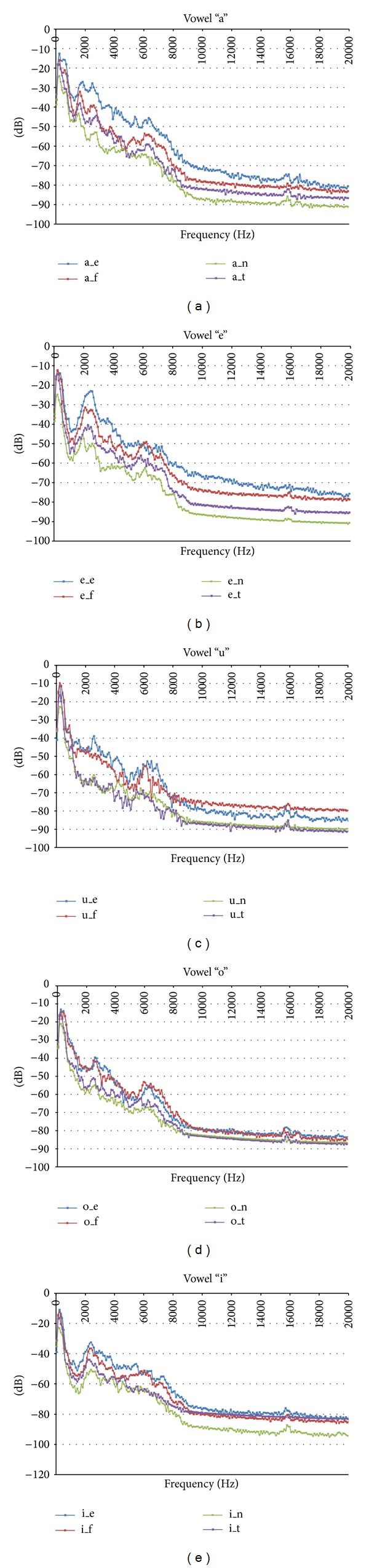
Average spectrum for all emotion-specific vowels across all male speakers. Description of identifiers: _e = anger, _f = happiness, _n = neutral, and _t = sadness.

**Figure 3:**
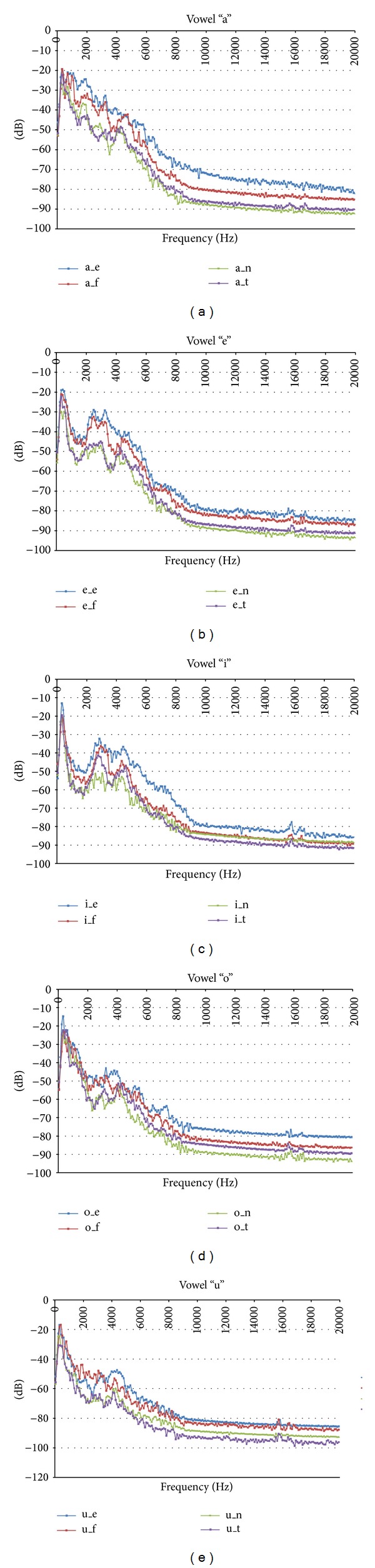
Average spectrum for all emotion-specific vowels across all female speakers. Description of identifiers: _e = anger, _f = happiness, _n = neutral, and _t = sadness.

After all spectrum plots were obtained for all samples of all vowels in the speech corpus, the average spectrum per gender and emotion was computed. In Figure 2 the average spectrum for all emotion-specific vowels across all male speakers is presented. The same concepts are presented in Figure 3 for the female speakers. Note the differences in the spectrum for all vowels depending on the emotion. These results are similar to the ones presented in [28]. Thus, the developed speech corpora is representative of the considered emotions and can be used for classification tasks.

In this work it is considered that by means of acoustic modelling of the emotion-specific vowels, an ASR system built with these models can be used to estimate emotional states. Hence, the state of a spoken sentence can be performed by counting the number of emotion-specific vowels found in the ASR's output. Note that with this approach, if a phoneme lexicon is added, information about the words spoken with a particular emotion can also be estimated. However, in order to perform this development, a suitable feature extraction method must be implemented. This is presented in the following section.

### 2.2. Feature Extraction

As commented in [3], there are no established analytical methods in the field of voice analysis that can reliably determine the intended emotion carried by the speech signal. However, in this field the spectral features obtained with Mel-frequency cepstral coefficients (MFCCs) have ben used with important results [3, 13]. MFCCs have been widely used in speech recognition because of superior performance over other features. These cepstrum-related spectral features have also been found to be useful in the classification of stress in speech [27, 33].

The Mel-frequency cepstrum is a representation of the short-term power spectrum of a sound, based on a linear cosine transformation of a log power spectrum on a nonlinear Mel scale of frequency [3]. MFCCs are based on the known variation of the human ear's perception to different frequencies, which can be expressed in the Mel-frequency scale [34, 35]. The coding of the emotional speech corpus was performed with the *HCopy* module of the Hidden Markov Model Toolkit (HTK) developed by the Cambridge University Engineering Department in the United Kingdom [34]. Details about the MFCC codification process can be found in [34, 35].

**Figure 4:**
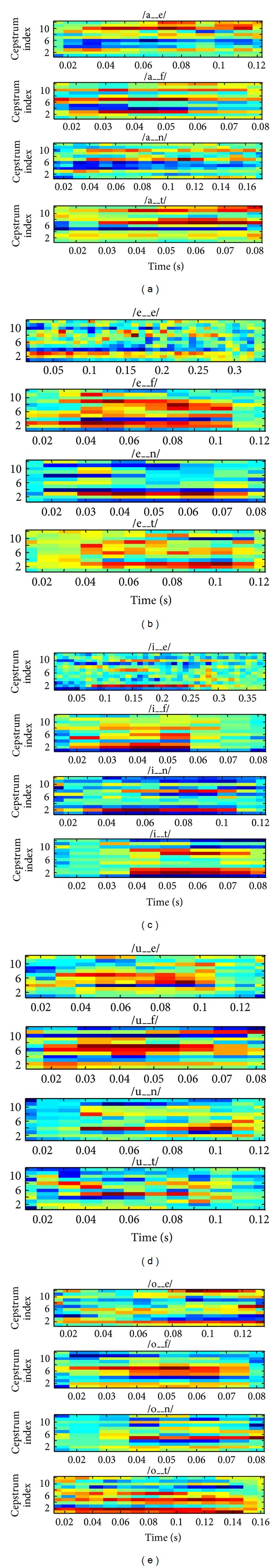
MFCCs obtained for some emotion-specific vowels of a male speaker from the built emotional speech database. Description of identifiers: _e = anger, _f = happiness, _n = neutral, and _t = sadness. HTK coding parameters: TARGETKIND = MFCC_E_D_A, WINDOWSIZE = 250000.0, USEHAMMING = T, PREEMCOEF = 0.97, NUMCHANS = 26, CEPLIFTER = 22, and NUMCEPS = 12.

For this work, 12 cepstral coefficients plus energy (E), delta (D), and acceleration (A) coefficients were computed [34]. In Figure 4 some examples of the MFCCs obtained for the vowels of one of the male speakers from the emotional speech database are shown with the parameters used for the codification tool *HCopy*. This corroborates the information presented in Figures 2 and 3, showing that features extracted with MFCCs can be used to identify emotional states given the differences presented in Figure 4.

## 3. Classification: Recognition Method

The approach of this work is that by means of acoustic modelling, particularly of the vowels, speech emotion recognition can be performed. Among the most common methods for acoustic modelling and classification the following can be mentioned: Vector Quantization (VQ), Gaussian Mixture Density (GMD) Models, Support Vector Machines (SVM), Artificial Neural Networks (ANNs), and Hidden Markov Models (HMMs) [3, 4, 13, 16, 17, 19, 27, 36].

In [4] VQ, ANNs, and GMD were trained with the speech features extracted from different sections of whole emotional sentences, obtaining short- and long-term features. The classification method determined the emotion from a particular sentence. This applied to the work presented in [3, 7, 16]. Because global features were considered by these works, specific features as those of vowels were not fully considered for modelling.

A work that considered this situation was the phoneme-class approach presented in [27]. In that work, two sets of HMM classifiers were built: a generic set of “emotional speech” HMMs (one for each emotion, as in common approaches) and a set of broad phonetic-class-based HMMs for each emotion type considered. Five broad phonetic classes (vowel, glide, nasal, stop, and fricative sounds) were used to explore the effect of emotional “coloring” on different phoneme classes. It was found that spectral properties of vowel sounds were the best indicator of emotions in terms of the classification performance.

Instead of building different HMM classifiers as in [27], with the proposed approach just a single HMM classifier is required. For this classifier, an HMM is built for each of the 28 phonemes in the Mexican Spanish language (23 consonants and 5 vowels) [30]. However, since each vowel can be associated with four emotions, the number of vowels is extended to 20 as presented in Table 6.

**Figure 5:**
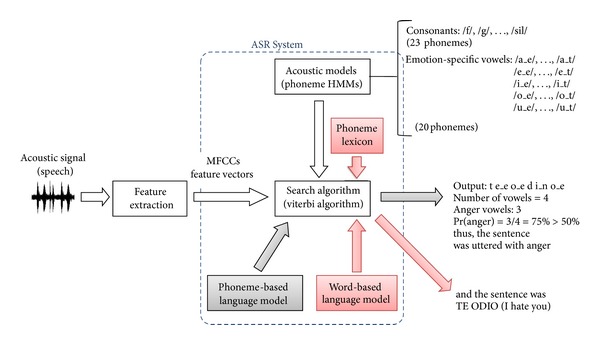
Structure of the ASR system for emotion recognition.

An Automatic Speech Recognition (ASR) system built with these HMMs would output phoneme sequences (including the emotion-specific vowels) when tested with emotional speech. A decision about the emotion present in the speech then can be performed by computing the frequency of emotion-specific vowels in the ASR's output. An advantage of using the ASR system is that, by incorporating a phoneme lexicon, word output sequences can be obtained, providing additional information about the sentence spoken with a particular emotion. The proposed HMM ASR system for emotion recognition is presented in Figure 5. Its elements were implemented with the HTK tool [34] and the details are presented in the following sections.

### 3.1. Acoustic Models

Hidden Markov Models (HMMs) were the method used for the acoustic modelling of the Mexican Spanish phonemes presented in Tables 5 and 6 (which include the emotion-specific vowels). The HMMs had the standard three-state left-to-right structure presented in [34] for acoustic modelling of phonemes with six Gaussian mixture components per state.

### 3.2. Phoneme Lexicon

The phonetic lexicon was built at the same time as the phonetic labelling of the speech corpus. A lexicon consists of a list of word entries with their respective phonetic transcription based on a given alphabet. For the initial phonetic labelling and creation of the lexicon of the emotional speech corpus, the word transcriptor *TranscribEMex * [37] was used. This tool was developed to phonetically label the DIMEX corpus for Mexican Spanish [37] using the updated Mexbet alphabet [30] presented in Table 5. Then, for the final phonetic labelling and creation of the lexicon, the identifiers _e, _f, _n, and _t were added to the vowel labels obtained with TranscribEMex according to the emotion of the speech.

Because in practice any word can be spoken with any emotion, the words in the system's lexicon also had an identifier associated with the emotion (_E, _F, _N, _T; see Figure 1) and each word was considered to be spoken with all emotions. Thus, for the word CASA (home) the lexicon had the following entries:

- CASA_E k a_e s a_e,
- CASA_F k a_f s a_f,
- CASA_N k a_n s a_n,
- CASA_T k a_t s a_t.

This has the possible outcome to recognize the emotion on each word in a sentence. During the ASR process, the lexicon restricts the phoneme sequences decoded by the search algorithm to form valid words. An example of this process is presented in Figure 5. The ASR system would produce the phoneme sequence /t e_e o_e d i_n o_e/ with a phoneme-based language model. When adding the lexicon the phoneme sequence gets restricted to form words. And then, by adding a word based language model, these words get restricted to form phrases. In this case, /t e_e/ = TE and /o_e d i_n o_e/ = ODIO by maximum likelihood.

### 3.3. Language Models

A language model represents the rules or probabilities that provide information about the valid phoneme/word structures in a language. For this work, bigram language models (2 grams) were estimated from the phoneme and word (orthographic) transcriptions of the corpus. Because as presented in Section 3.2 each word in the speech corpus was considered to be spoken with all emotions, in total four phonetic and word transcriptions of the corpus were considered for language model estimation.

The following HTK modules were used for this purpose.

- *HLStats* was used to compute label statistics for the purpose of estimating probabilities of occurrence of each single phoneme/word in the corpus (unigram probabilities). If configured to estimate bigram probabilities, it provides the associated probabilities of occurrence for the different pairs of phonemes/words found in the phonetic and orthographic transcriptions. For unseen pairs of words, *backed-off* bigram probabilities can be estimated from the unigram probabilities [34].
- *HBuild* was used to build a phoneme/word network with the statistics estimated with *HLStats*. This module generated the statistical language model for the ASR system.

### 3.4. Search Algorithm

Speech recognition was performed with the Viterbi algorithm implemented with the module *HVite* of HTK. This module takes as input the coded speech to be recognized, the network describing the allowable phoneme/word sequences in the language (given by the language model), the lexicon, and the set of trained HMMs.

## 4. Performance

**Figure 6:**
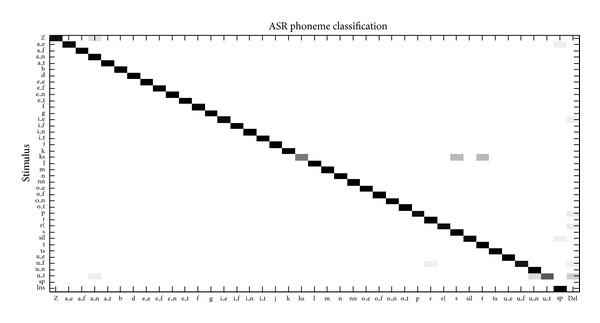
Classification performance of the ASR system for speech recognition on the speech corpus (training = testing set).

Initially the HMM ASR classifier was trained and tested with all the speech samples from the six speakers (three males: MS1-3, three females: FS1-3) of the emotional speech corpus. The phoneme confusion matrix of this test is presented in Figure 6. As observed, there are very few insertion (Ins), deletion (Del), and substitution errors in the recognized speech. The confusions between the emotion-specific vowels are minimal, and this is a significant result because accurate levels of classification of these phonemes are required for emotion recognition. For assessment of recognition accuracy the equation:

(1) Accuracy=L−Del−Subst−InsL×100,

was used, where *L* is the number of elements (phonemes/words) in the reference transcription of the recognized speech and Subst is the number of substitutions (phonemes/words) present in the ASR's output. For the ASR's phoneme output presented in Figure 6 the following statistics were obtained: *L* = 3916, Del = 152, Ins = 30, and Subst = 148, leading to a phoneme recognition accuracy of 91.57%. In contrast, for the ASR's word output an accuracy of 90.59% was obtained. Both types of performance are normal when testing is performed on training sets. The associated emotion recognition results considering the frequency of emotion-specific vowels are presented in Table 7. For all emotions, recognition performance is higher or equal to 95%.

After the initial test, the system was tested for each speaker in the following way:

1. a speaker is selected randomly (i.e., MS1);
2. the HMMs of the ASR system are trained/built with the speech data of the other speakers (i.e., MS2-3, FS1-3);
3. from the speech data of the selected speaker (i.e., MS1), four randomly selected sentences per emotion are taken for speaker adaptation. In this case, Maximum Likelihood Linear Regression (MLLR) [34] was used as the adaptation technique;
4. phoneme-based ASR is performed with the remaining speech data (six sentences per emotion) of the selected speaker (i.e., MS1). With this adaptation/testing scheme more data is available for evaluation of the system in comparison with other works as in [4] where approximately 75% of recorded sentences were used for training and 25% for testing;
5. vowel and identifier counting is performed on the recognized speech sentences. The identifier with more presence in the vowels found in the ASR's phoneme output (a threshold of 50% was set) determines the dominant emotion in the speech sentence (_e for anger, _f for happiness, _n for neutral, and _t for sadness);
6. repeat from step (1) until all speakers are selected.

The process described previously was iterated five times in order to obtain different random sets of adaptation and testing sentences per speaker. In Table 8 the details of the emotion recognition results for the individual speakers are presented across the five iterations. Note that for some cases the percentages of word and phoneme recognition accuracy are not as high as the emotion recognition percentages. This is because the accuracy statistics consider all phonemes (see (1)) which consists of vowels and consonants, and emotion is determined based on only vowels. Also, during the word ASR process, for each single word there are four possible choices (see Section 3.2) and thus uncertainty is higher when compared with a standard process.

In Table 9 the average performance and total performance for each speaker (and all speakers) are presented. This data is computed from the results presented in Table 8. As presented, the emotions that are more consistently identified (with a very small standard deviation) are neutral and sadness with 98.89% and 100.00%, respectively. The identification of anger and happiness shows slightly more inconsistencies with a standard deviation of 12.47 and 13.75 although the average recognition is 87.02% and 91.39%, respectively. In this case, significant confusions between these two emotions were found. This situation was observed also in [4, 15, 16, 27]. Nevertheless, for recorded speech data, these results are over the 85% reported by other works with similar number of emotions [16, 27].

## 5. Conclusions and Future Work

In this paper the development of an emotional speech corpus for Mexican Spanish and an approach for emotion recognition based on acoustic modelling of vowels was presented. HMMs were used for the modelling of consonants and emotion-specific vowels, and these were integrated into an ASR system to generate phoneme and word sequences with emotion identifiers. With this approach the following average recognition results were obtained: 87.02% for anger, 91.39% for happiness, 98.89% for neutral, and 100% for sadness.

Some situations presented by other works were observed in this work. For example, the spectrum differences in vowels give the emotional status [28] and some confusions between anger and happiness [15, 16, 27]. Thus, the speech corpus presented similar outcomes as other databases used in the field of emotion recognition.

An advantage of the emotion recognition approach presented in this paper is that it shares the building stages of a general purpose ASR system. Thus, considering that a labelled emotional speech corpus is available, the implementation of the emotion recognizer can be performed quickly. In comparison with other works as [27] only one classifier is required and emotion recognition can be performed over whole sentences or for each word in a sentence.

Also, standard adaptation techniques can be used to make the ASR usable for other speakers for the same purpose of emotion recognition. In this case, the new speaker would be required to produce emotional speech to perform adaptation. For this task, the stimuli presented in Tables 1–4 can be used, and automatic labelling of the adaptation speech can be performed with an emotion-adapted phoneme transcription tool. Online speaker adaptation with the option to add vocabulary to an existing ASR system was presented in [38] with significant results for disordered speech. This can be explored for the case of emotional speech recognition, and ongoing work is focused on the following points:

- to test the approach with other emotional speech databases with more emotions;
- to increase the vocabulary and speakers in the Mexican Spanish emotional database. This is important to test emotion recognition performance with a larger vocabulary and a more complex language model;
- to build an ASR with the proposed approach to recognize spontaneous emotional speech. In this case, online (automatic) speaker adaptation with live emotional speech is required;
- to compare the performance of the proposed approach when other classification techniques as SVM and ANNs are considered;
- to improve current performance.

## Figures and Tables

**Table 1:** Emotional stimuli: “enojo” (anger).

No	Sentence
1	Tu siempre llegas tarde (You always arrive late)
2	Voy a matarte (I am going to kill you)
3	No vengas aquí (Do not come here)
4	Ya cállate (Shut up)
5	Si no te gusta hazlo tú (If you do not like it then do it yourself)
6	Qué quieres que te diga (What do you want me to tell you)
7	Eres un sinvergüenza (You are a shameless)
8	Me las vas a pagar María Fernanda (You are going to pay, Maria Fernanda)
9	No te quiero volver a ver nunca más (I do not want to see you ever again)
10	Te voy a poner de patitas en la calle (I will take you out of the house)

**Table 2:** Emotional stimuli: “felicidad” (happiness).

No	Sentence
1	Hey, pasé el exámen (Hey, I passed the exam)
2	Ya tengo a mi hijo en casa (I finally got my son to come home)
3	Tengo una gran familia (I have a great family)
4	Me encanta que mis hijos me vengan a visitar (I love that my children come to visit me)
5	El canto es mi vida, me encanta cantar (Singing is my life, I love to sing)
6	Gané un millón de dólares (I won a million dollars)
7	Por fin se pagó la casa (Finally, the house was paid)
8	Mañana es mi fiesta (Tomorrow is my party)
9	Gracias papá por mi ropa nueva (Thank you dad for my new clothes)
10	El bebé nació tan saludable (The baby was born very healthy)

**Table 3:** Emotional stimuli: “neutro” (neutral).

No	Sentence
1	El cielo está nublado (The sky is cloudy)
2	La casa es rosada (The house is pink)
3	El comedor es para seis personas (The dining room is for six people)
4	Mañana vamos a la escuela (Tomorrow we will go to school)
5	Crucemos la calle (Let's cross the street)
6	Tengo mucha tarea (I have a lot of homework)
7	La guitarra de mi mamá está sucia (My mom's guitar is dirty)
8	Mi niño juega sus juguetes nuevos (My kid plays his new toys)
9	Es enorme tu casa (Your home is huge)
10	Es mejor la leche entera que la deslactosada (Whole milk is better than lactose-free milk)

**Table 4:** Emotional stimuli: “tristeza” (sadness).

No	Sentence
1	Me siento tan mal (I feel so bad)
2	Mi perrito murió (My little dog died)
3	Juan Antonio, porqué me haz engañado? (Juan Antonio, why you cheated on me?)
4	Digame que mi hijo está bien doctor (Please doctor, tell me if my son is alright)
5	Los ricos también lloran, pero los pobres lloramos más (The rich also cry, but we, the poor, cry more)
6	Espero que algún dia encontremos a mi hijo (One day I hope to find my son)
7	Mamá, porqué ya no viene mi papá? (Mom, why my father is no longer coming?)
8	Nadie me quiere porque soy fea (Nobody likes me because I am ugly)
9	De qué me sirve el dinero si no la tengo a mi lado? (Money is of no use if I do not have her by my side)
10	Gasté todo en bebida, ahora no me queda más que mi desilusión (I spent all my money in alcohol, now I only have my disappointment)

**Table 5:** IPA and Mexbet representation of the Mexican Spanish phonemes [30].

Description	IPA	Mexbet
Voiceless bilabial stop	p	p
Voiceless dental stop	t	t
Voiceless velar stop	k	k
Voiced bilabial stop	b	b
Voiced dental stop	d	d
Voiced velar stop	g	g
Voiceless palatal affricate	$\overset{⌢}{ʧ}$	tS
Voiceless labiodental fricative	f	f
Voiceless alveolar sibilant	s	s
Voiceless velar fricative	x	x
Voiced palatal fricative	*Ӡ*	Z
Bilabial nasal	m	m
Palatal nasal	*ɲ*	ñ
Alveolar nasal	n	n
Alveolar lateral	l	l
Alveolar trill	r	r
Alveolar flap	*ɾ*	r(
Close front unrounded vowel	i	i
Close-mid front unrounded vowel	e	e
Open front unrounded vowel	a	a
Close-mid back rounded vowel	o	o
Close back rounded vowel	u	u

**Table 6:** Emotion-specific vowels in the emotional stimuli.

Emotion	Vowel/phoneme	Frequency
Anger	a_e	31
	e_e	28
	i_e	11
	o_e	9
	u_e	6

Happiness	a_f	41
	e_f	28
	i_f	18
	o_f	12
	u_f	4

Neutral	a_n	38
	e_n	32
	i_n	7
	o_n	14
	u_n	10

Sadness	a_t	32
	e_t	42
	i_t	26
	o_t	34
	u_t	4

**Table 7:** Classification performance of the ASR system for emotion recognition on the speech corpus (training = testing set).

	Anger	Happiness	Neutral	Sadness
Anger	95.00	2.50	2.50	0.00
Happiness	0.00	95.24	4.76	0.00
Neutral	0.00	0.00	100.00	0.00
Sadness	0.00	0.00	5.00	95.00

**Table 8:** Classification performance of the ASR system for speech and emotion recognition (individual speakers from the speech corpus) across five iterations.

Speaker	Emotion	Iteration 1	Iteration 2	Iteration 3	Iteration 4	Iteration 5
MS1	Anger	100.00	100.00	100.00	100.00	100.00
	Happiness	66.67	66.67	66.67	66.67	66.67
	Neutral	100.00	100.00	100.00	100.00	100.00
	Sadness	100.00	100.00	100.00	100.00	100.00
	Word accuracy	75.00	78.75	80.00	80.00	80.00
	Phoneme accuracy	80.12	82.40	83.46	84.67	83.46

MS2	Anger	71.43	71.43	83.33	83.33	100.00
	Happiness	100.00	83.33	75.00	100.00	75.00
	Neutral	100.00	100.00	100.00	100.00	100.00
	Sadness	100.00	100.00	100.00	100.00	100.00
	Word accuracy	78.26	86.34	88.20	87.58	92.55
	Phoneme accuracy	80.79	88.35	90.02	88.80	93.65

MS3	Anger	83.33	83.33	100.00	83.33	100.00
	Happiness	100.00	100.00	100.00	100.00	75.00
	Neutral	100.00	100.00	100.00	100.00	100.00
	Sadness	100.00	100.00	100.00	100.00	100.00
	Word accuracy	91.30	89.44	98.76	93.79	92.55
	Phoneme accuracy	90.77	91.38	98.49	94.86	93.65

FS1	Anger	100.00	83.33	100.00	100.00	100.00
	Happiness	100.00	100.00	100.00	100.00	100.00
	Neutral	100.00	100.00	66.67	100.00	100.00
	Sadness	100.00	100.00	100.00	100.00	100.00
	Word accuracy	76.25	74.38	68.13	80.63	85.63
	Phoneme accuracy	82.28	81.83	75.83	83.63	88.14

FS2	Anger	62.50	83.33	85.71	66.67	83.33
	Happiness	100.00	100.00	100.00	100.00	100.00
	Neutral	100.00	100.00	100.00	100.00	100.00
	Sadness	100.00	100.00	100.00	100.00	100.00
	Word accuracy	74.38	82.50	85.63	88.13	87.50
	Phoneme accuracy	77.13	87.04	87.80	89.33	88.11

FS3	Anger	85.71	62.50	71.43	83.33	83.33
	Happiness	100.00	100.00	100.00	100.00	100.00
	Neutral	100.00	100.00	100.00	100.00	100.00
	Sadness	100.00	100.00	100.00	100.00	100.00
	Word accuracy	88.75	74.38	92.50	79.38	88.75
	Phoneme accuracy	89.33	79.57	92.23	80.79	90.40

**Table 9:** Average classification performance of the ASR system for speech and emotion recognition (individual speakers from the speech corpus) across five iterations.

Speaker	Statistic	Anger	Happiness	Neutral	Sadness	Word accuracy	Phoneme accuracy
MS1	Average	100.00	66.67	100.00	100.00	78.75	82.82
	Std Dev	0.00	0.00	0.00	0.00	2.17	1.71

MS2	Average	81.90	86.67	100.00	100.00	86.59	88.32
	Std Dev	11.74	12.64	0.00	0.00	5.21	4.70

MS3	Average	90.00	95.00	100.00	100.00	93.17	93.83
	Std Dev	9.13	11.18	0.00	0.00	3.52	3.09

FS1	Average	96.67	100.00	93.33	100.00	77.00	82.34
	Std Dev	7.45	0.00	14.91	0.00	6.59	4.41

FS2	Average	76.31	100.00	100.00	100.00	83.63	85.88
	Std Dev	10.85	0.00	0.00	0.00	5.61	4.96

FS3	Average	77.26	100.00	100.00	100.00	84.75	86.46
	Std Dev	9.96	0.00	0.00	0.00	7.56	5.85

Total (all speakers)	Average	87.02	91.39	98.89	100.00	83.98	86.61
	Std Dev	12.47	13.75	6.09	0.00	7.30	5.55
